# Supplementary material for: miR-27a-5p Attenuates Hypoxia-induced Rat Cardiomyocyte Injury by Inhibiting Atg7
Source: Int J Mol Sci. 2019 May 16;20(10):2418. doi: 10.3390/ijms20102418 (PMC6566783; doi:10.3390/ijms20102418)
Supplement: Supplementary file 1 [file ijms-20-02418-s001.pdf]

Table S1. Overview of BP changes in different ligation timepoints

Ligation timpoints	0 h	1 h	2 h	3 h	4 h	5 h	6 h
Mean (Sham)	105.35	94.55	89.82	90.43	86.75	96.4	96.17
Mean (AMI)	102.22	67.97	71.08	59.32	54.22	46.02	43.39
SD (Sham)	6.05	8.41	5.46	8.20	7.54	6.28	11.75
SD (AMI)	5.22	7.79	4.26	10.14	9.05	11.98	12.15
Cohen's d	0.5539567	3.2790809	3.8269133	3.3737619	3.9054956	5.2674025	4.416118
Power	0.1975046	0.9999997	1	0.9999988	1	1	1

Note: BP, blood pressure; Cohen's d is used to evaluate the effect size.

Table S2. Organ index of main visceral tissues (%)

Organ index	Heart	Liver	Spleen	Lung	Kidney
Sham	0.309 ± 0.003	3.897 ± 0.177	0.297 ± 0.033	0.439 ± 0.046	0.939 ± 0.173
AMI	0.295 ± 0.020*	3.889 ± 0.496	0.225 ± 0.034 **	0.510 ± 0.038	0.857 ± 0.042

Note: AMI, acute myocardial infarction; *$p < 0.05$; ** $p < 0.01$.

Table S3. Validation reporting for miR-27a-5p-*Atg7* interaction

1	miRNA	Gene name	Mir27a
		Entrez ID	100314006
2	Target gene	Gene name	Atg7
		Entrez ID	312647
		Transcript (if available)	*Atg7*-201 (ENSRNOT00000067532.2)
3	Species	Species	Rattus norvegicus
		Species ID	10116
4	Experimental validation of miRNA–target interaction	Sequence of the target region, 5'-3'	GCCCT
		Genomic location of MTI / 3'UTR	4:146776236-146776240 / 3'UTR: 25-29
		Method for experimental validation	luciferase reporter assay, qPCR, western blot
		Tissue, cell lines	heart; H9c2 cell lines
5	Sequence variant	rs number (synonym)	na
6	Associated disease or phenotype	As named in the reference	Acute myocardial ischemia; hypoxia treatment
		DOID (if available)	na
7	Reference	Author, year	na
		PMID	na

Note: na, not available.

Table S4. The specific primers used for qRT-PCR

Gene name	Accession	Sequence (5' to 3')
rno-miR-27a-5p	MIMAT0004715	F: AGGGCTTAGCTGCTTGTGAGCA<br>R: Uni-miR qPCR Primer, included in kit
Caspase-3	NM_012922.2	F: CATGGCCCTGAAATACGAAGTC<br>R: GCAGGCCTGAATGATGAAGAGTTT
P53	NM_030989.3	F: CTCCTCTCCCCAGCAAAAGA<br>R: GTAGACTGGCCCTTCTTGGT
Faslg	NM_012908.1	F: GCCCGTGAATTACCCATGTC<br>R: TAGTGGTGATGGAGGTGGTG
BAX	NM_017059.2	F: TGGCCTCCTTTCCTACTTCG<br>R: AAAATGCCTTTCCCCGTTCC
Bcl-2	NM_016993.1	F: GACGCGAAGTGCTATTGGT<br>R: TCAGGCTGGAAGGAGAAGAT
Atg7	NM_001012097.1	F: GCTGGTCTCCTTGCTCAAAC<br>R: CAGGGTGCTGGGTTAGGTTA
GAPDH	NM_017008.4	F: AACGACCCCTTCATTGACCTC<br>R: CCTTGACTGTGCCGTTGAACT
U6	---	F: CTCGCTTCGGCAGCACA<br>R: AACGCTTCACGAATTTGCGT
rno-miR-27a-5p mimics		5'-AGGGCUUAGCUGCUUGUGAGCA-3'<br>3'-UGCUCACAAGCAGCUAAGCCCU-5'
rno-miR-27a-5p inhibitor		5'-UCCCGAAUCGACGAACACUCGU-3'
Negative control		Universal sequences (Ribobio, Guangzhou, China)

Note: *GAPDH* and *U6* were used as housekeeping genes to normalize mRNA and miRNA, respectively.

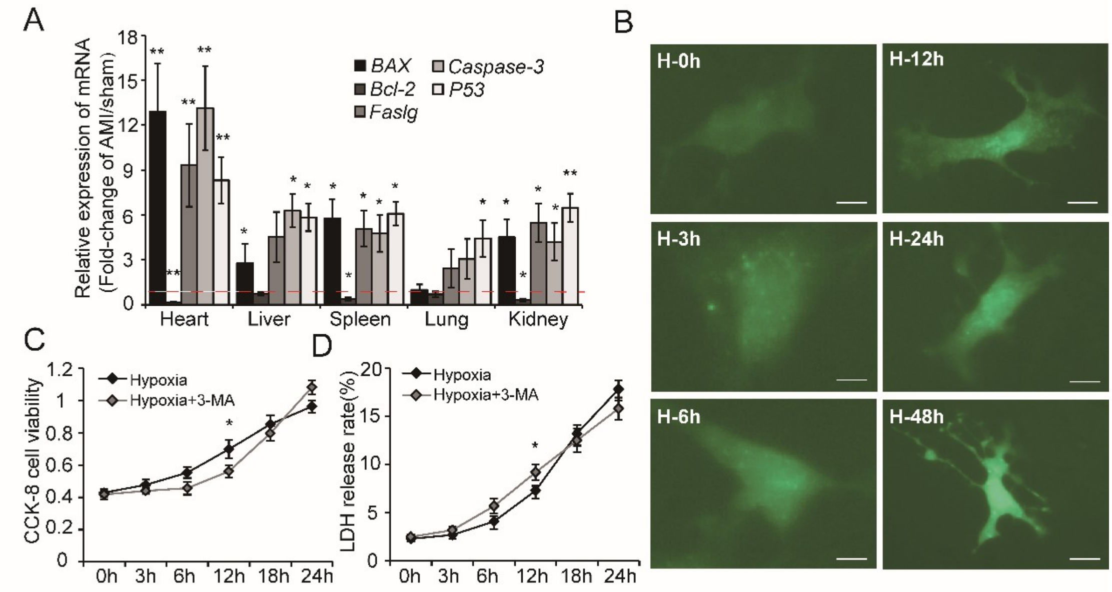

Figure S1. (**A**) The expression level of apoptosis-related gene in main visceral tissues were evaluated using qRT-PCR in in sham and AMI rats. (**B**) H9c2 cells were transfected GFP-LC3 plasmids and then exposed to hypoxia at different timepoints, fluorescent punctae were observed by a confocal fluorescence microscope; scale bar: 5μm. H9c2 cells were preincubated 3-MA (10 mM) for three hours and then exposed to hypoxia at different time. Cell viability (**C**) and membrane damage (**D**) were detected by CCK-8 assay and LDH release assays, respectively. Three independent experiments were performed in triplicate. Data are expressed as the mean ± SD. * $p < 0.05$, ** $p < 0.01$. H: hypoxia.
